# Dose Recommendations for Drugs in Patients With Liver Cirrhosis (The ALIVe Study): Protocol for a Multiphase Validation and Consensus Study

**DOI:** 10.2196/89042

**Published:** 2026-06-09

**Authors:** Katharina Karsten Dafonte, Philipp Lutz, Christian Jansen, Christian P Strassburg, Robert Németh, Gunther Hartmann, Ulrich Jaehde, Martin Coenen

**Affiliations:** 1 Institute of Clinical Chemistry and Clinical Pharmacology, University Hospital Bonn, Venusberg-Campus 1, Bonn, 53127, Germany, 49 22828714036; 2 Department of Internal Medicine I, University Hospital Bonn, Bonn, Germany; 3 Institute for Medical Biometry, Informatics and Epidemiology, Medical Faculty, University of Bonn, Bonn, Germany; 4 Department of Clinical Pharmacy, Institute of Pharmacy, University of Bonn, Bonn, Germany

**Keywords:** liver cirrhosis, medication safety, drug safety, drug recommendations, dose adjustment, drug selection, Child-Pugh classification, pharmacist-led intervention, pharmacist

## Abstract

**Background:**

Liver cirrhosis leads to an impaired liver function with reduced metabolic capacity, which affects the pharmacokinetics of several drugs, requiring dose adjustments. Although limited literature provides guidance on appropriate administration of drugs in cirrhosis, no guidelines currently exist for dose selection or adjustment.

**Objective:**

The objective of this study is to provide guidance on the selection, dosing, and appropriate use of drugs in patients with liver cirrhosis and evaluate the clinical application of these recommendations.

**Methods:**

Three steps are planned to establish dose recommendations for patients with liver cirrhosis. First, a systematic literature search will be conducted to identify specific recommendations for drug selection and dosing in cirrhosis, and the literature will be assessed for reporting quality and evidence level. Subsequently, the resulting recommendations will undergo an internal preassessment procedure for relevance of the covered drugs with regard to availability, the clinical impact of adverse drug reactions, the frequency of use, and the expected benefit of dose adjustment. Second, a modified Delphi procedure will be conducted to (1) analyze the clinical handling of the identified drugs by experts in clinical practice in a first round and (2) parse changes resulting from the inclusion of medication-related information and further harmonize differing dose recommendations in a second round. Finally, the adopted dose recommendations will be implemented in a clinical study involving patients with liver cirrhosis to analyze the impact of the recommendations on patient safety.

**Results:**

The planned clinical validation phase started in January 2025 and is currently underway. As of May 2026, 121 patients have been enrolled. Statistical analyses will be conducted in accordance with the predefined analysis plan. Findings from the literature review, the Delphi consensus process, and the clinical validation phase will be reported in subsequent publications. Final study results and data analysis are anticipated in August 2026.

**Conclusions:**

This protocol outlines a structured approach combining a systematic literature review of specific dose recommendations in patients with cirrhosis that integrates a quality assessment to ensure the inclusion of only high-quality evidence, expert opinions through a Delphi consensus aligning differing recommendations, and a clinical validation to support safer drug therapy in patients with liver cirrhosis.

## Introduction

Liver cirrhosis with advanced fibrotic remodeling of the parenchyma as the terminal stage of different chronic liver diseases leads to impaired liver function with reduced metabolization capacity, shunt circulation, and reduced serum protein levels [1,2]. It is classified into 3 grades of severity according to the Child-Pugh scale, which incorporates both objective laboratory (bilirubin, albumin, and international normalized ratio) and clinical (encephalopathy and ascites) parameters that are not easily quantifiable [2]. As the reduced metabolization capacity of the liver affects the pharmacokinetics of several drugs, their doses have to be adjusted. Missing dose adjustment or administration of contraindicated drugs are common medication-related problems (MRPs) [3,4]. It can be assumed that 33% of patients with cirrhosis experience at least one adverse drug reaction (ADR) during their hospital stay due to missing dose adjustments, which may result in a considerable prolongation of hospitalization in affected patients [5]. Of these ADRs, 68% are considered preventable [6]. There is plenty of literature that aims to assist in adequate administration of drugs in this population [7,8]. However, despite the availability of multiple reviews, consensus papers, and guidance documents addressing individual aspects of drug use in liver cirrhosis [6-8], no comprehensive clinical practice guideline for drug selection and dose adjustment across disease stages is currently available. As the Summary of Product Characteristics (SmPC) usually only provides vague information, dose adjustments in patients with hepatic impairment remain challenging in clinical practice [9].

To address this problem, we designed a systematic 3-step approach to identify dose recommendations for drugs in patients with liver cirrhosis via a systematic literature search, standardization of the recommendations using a Delphi consensus method, and direct application of the results to hospitalized patients with cirrhosis in a clinical study. The aim of this study is to provide support for the choice of an appropriate active substance within a therapeutic class (drug selection) and dose adjustment, as well as additional guidance for the use of drugs in cirrhosis. In contrast to the existing drug-specific safety classifications and database by Weersink et al [7], this study focuses on the harmonization of published dosing recommendations through a structured, multidisciplinary Delphi process and the subsequent real-world application of these consensus-validated recommendations in a prospective, pharmacist-led clinical intervention.

## Methods

### Overview

**Figure 1.**
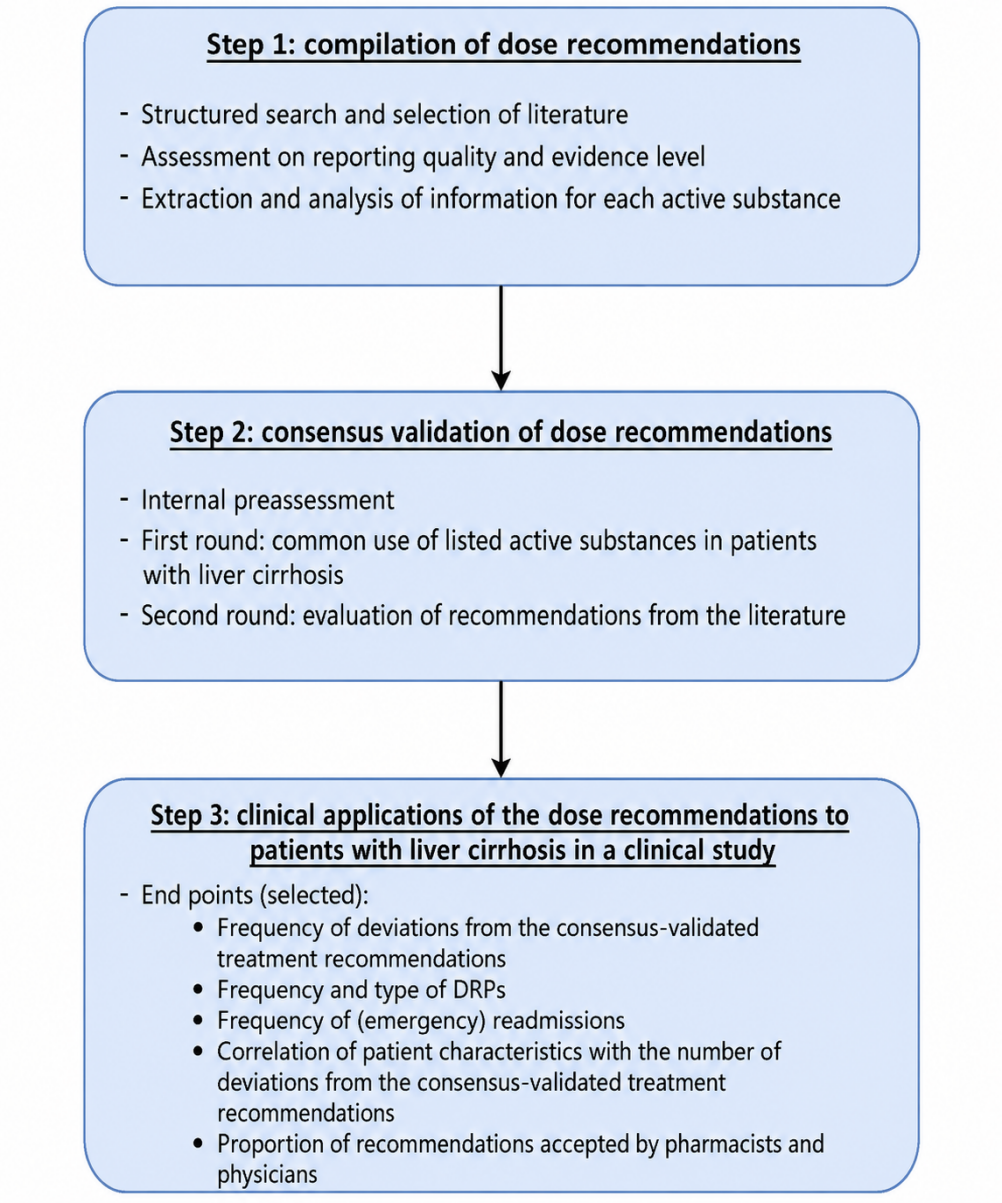
Flowchart of the process: transferring dose recommendations from the literature to clinical application. DRP: drug-related problem.

Three steps are planned to transfer dose recommendations for drugs from the literature via consensus validation to clinical application in patients with liver cirrhosis (Figure 1). The different study components are reported in accordance with established reporting guidelines. The systematic literature phase is mapped to the PRISMA (Preferred Reporting Items for Systematic Reviews and Meta-Analyses) 2020 guidelines; the Delphi consensus process is mapped to the CREDES (Guidance on Conducting and Reporting Delphi Studies) checklist; and the clinical validation phase is mapped to the SPIRIT (Standard Protocol Items: Recommendations for Interventional Trials) 2013 guidelines, including the SPIRIT-Outcomes extension.

### Compilation of Dose Recommendations

#### Structured Search and Selection of the Literature

The details of the identification of relevant evidence on dosing recommendations for patients with liver cirrhosis via a systematic literature review, including the search strategy, screening workflow, and inclusion and exclusion criteria, have recently been published [10]. In brief, structured searches of PubMed and Embase were conducted to identify peer-reviewed publications providing explicit dosing recommendations for patients with liver cirrhosis (Textbox 1). Only publications offering specific and clinically actionable guidance beyond the SmPC were considered eligible. This approach focused on already formulated dosing guidance rather than on isolated primary pharmacokinetic studies to ensure comparability across substances and alignment with real-world prescribing practice.

Textbox 1. **Original search strings for the PubMed and Embase databases used in the structured literature search.**

**Search string 1**

Search: ((liver) OR (hepatic)) AND ((impairment) OR (dysfunction) OR (damage) OR (injury)) AND ((dose) OR (dosage)) AND ((recommendation) OR (adjustment) OR (modification) OR (adaptation))

**Search string 2**

Search: (recommendation) AND (drug) AND ((prescription) OR (dosage) OR (safety)) AND ((liver) OR (hepatic)) AND ((cirrhosis) OR (disease) OR (impairment))

#### Assessment of Reporting Quality and Evidence Level

Reporting quality was evaluated to determine whether the derivation of dosing recommendations was sufficiently transparent and reproducible. Evidence levels were assigned according to the Oxford Centre for Evidence-Based Medicine framework [11]. Reporting checklists (PRISMA for systematic reviews [12], AGREE [Appraisal of Guidelines for Research and Evaluation] for guidelines [13], and CREDES for Delphi procedures [14]) were applied to assess transparency and reproducibility of reporting rather than intrinsic scientific validity or clinical relevance. Publications were excluded only in cases of major reporting deficiencies that prevented understanding or reproduction of how dose recommendations were derived. References with minor or moderate reporting limitations were retained, and their reporting shortcomings were documented with the assigned evidence level [10].

#### Extraction and Analysis of Information for Each Active Substance

From the included publications, only recommendations allowing for direct clinical application (eg, clear dose adjustments, interval modifications, avoidance statements, or specification of no adjustment required) were considered. Drug classes requiring highly individualized and indication-specific dosing decisions (eg, cytostatics) were excluded as treatment protocols for these agents are typically governed by specialized oncology guidelines and do not permit generalized dose recommendations across patient groups. The extracted recommendations were structured according to active substance and, where available, stratified using the Child-Pugh classification [15,16] to enable direct comparison and consensus validation. Where multiple publications provided differing recommendations for the same active substance, statements were compared to assess consistency. The resulting structured compilation of dosing recommendations served as the basis for the subsequent Delphi consensus process. A detailed description of the comparative methodology is available in the original systematic review [10].

### Definition of Consensus-Validated Dose Recommendations

#### Internal Preassessment Concerning the Clinical Relevance of Expected ADRs

As the systematic literature search was carried out without restrictions on the publication date, the identified active substances will be cross-checked for relevance against the stock list of the pharmacy of University Hospital Bonn, including all drugs and medical devices used by the wards of the Department of Internal Medicine I. Active substances not included in the pharmacy stock list of this hospital of maximum care will be excluded from further processing as their absence indicates that they are not used regularly in clinical practice. However, before the medications are removed, their clinical relevance will be checked again by 2 pharmacists.

For feasibility reasons, and to focus on active substances particularly relevant in cirrhosis, a preselection will be conducted through an internal preassessment procedure to rate (1) the relevance of dose adjustments of each active substance considering the severity of potential ADRs mentioned in the literature, (2) the frequency of prescription particularly in patients with cirrhosis, and (3) the expected benefit of dose adjustment to avoid possible side effects. The expert panel will consist of 2 hepatologists and 1 clinical pharmacologist. Each expert will have specific expertise in the treatment of patients with liver cirrhosis. The following data from the selected literature will be the basis for the evaluation: (1) the authors’ recommendations and their justification; (2) further information from the references on hepatotoxicity and ADRs; (3) information from the corresponding SmPC on precautions, hepatotoxicity, and ADRs; and (4) information from the LiverTox database, assigned to category A, B, C, D, or E* in the Categorization of the Likelihood of Drug-Induced Liver Injury. For analysis of the assessments, the 3 different criteria (clinical relevance of ADRs, frequency of use, and expected benefit of dose adjustment) will be weighted in a ratio of 3:1:2. Considering the point scale from 0 to 3 provided to the experts, a maximum of 18 points per active substance can be achieved. Only the most relevant active substances rated, reaching a predetermined minimum score of 13.5 points, will be included for further processing. The cutoff is pragmatically predefined to focus on substances with the highest expected clinical impact and to improve the feasibility of the consensus evaluation.

#### Delphi Survey on the Dose Recommendations

The Delphi technique is a well-established method for the evaluation and assessment of controversial issues by experts, with the aim to produce reasonably reliable consensus-validated statements [14,17]. A Delphi procedure will be conducted that meets the three basic characteristics: (1) anonymity (the 2-round consensus process will be conducted anonymously; the list of participants will only be disclosed to the experts after the evaluation has been completed), (2) iteration (2 interlinked sequential rounds will be carried out), and (3) controlled feedback (in the second round, participants will receive both the consensus results from the first round and their own initial responses, allowing them to reconsider and, if necessary, adjust their assessments; in this way, convergence of evaluations from the first to the second round can be achieved). Unlike the classic Delphi approach, additional information will be provided to the experts in the second round, which they can incorporate into their assessments. This approach allows experts to reconcile their initial clinical judgments with available evidence and formal guidance.

A total of 12 experts with particular expertise in drug safety in hospitalized patients with cirrhosis and involved in drug decision-making processes in hospitals were recruited: 6 (50%) hepatologists, 3 (25%) clinical pharmacologists, and 3 (25%) clinical pharmacists. The assessment sheets will undergo pretesting in 2 rounds by a pharmacist, a clinical pharmacologist, and 2 hepatologists to identify any issues related to understanding and processing of the sheet, resulting in the final version.

According to the rules of the Association of the Scientific Medical Societies for structured consensus in Germany, a recommendation will be considered accepted if at least 75% of the voting experts agree; Table 1 provides a concise overview of the agreement thresholds and decision categories applied. In contrast, divergence will be assumed if (1) no majority consensus is reached, (2) a majority is achieved but at least one-third of the votes indicate a conflicting recommendation (eg, standard dose vs avoidance), and (3) the majority rejects the evidence but no consensus on an alternative approach can be reached (this item only applies to the second round).

**Table 1.** Grade of consensus.

Grade of consensus	Acceptance (%)	Experts (N=12), n
Strong consensus	≥96	12
Consensus	76‐95	10‐11
Majority approval	51‐75	7‐9
No majority approval	≤50	≤6

#### First Round of the Delphi Survey: Usual Prescription of the Drugs

The first round is intended to capture the usual prescribing practice concerning the choice of an appropriate active substance (drug selection) within a therapeutic class and dose adjustment of a selected drug. In this design, structured external evidence is not provided in the first round to capture routine expert prescribing behavior prior to evidence-informed reassessment in the second round. In this context, the experts will be asked about their common practice with the active substances in an average hospitalized patient with liver cirrhosis who does not have additional comorbidities that are not typically found in patients with cirrhosis. The experts will have the options to select, for each grade of the Child-Pugh classification, whether they usually (1) prescribe the unchanged dose as they do to patients without liver disease (no dose adjustment), (2) make a dose adjustment, or (3) avoid the drug completely. If they select a dose adjustment, the experts will be required to provide the type of dose adjustment (eg, dose reduction, prolongation of the dosing interval, or dose adaptation based on the results of therapeutic drug monitoring [TDM]). A comment field will be provided for each active substance, allowing experts to describe any limitations and specific considerations that appear relevant to them (for the standardized response options used to capture routine prescribing practice, see Multimedia Appendix 1). This assessment yields a valid and unbiased baseline, is itself an objective of the study, and will provide clinically meaningful data on existing prescribing behavior.

#### Second Round of the Delphi Survey: Selection of the Dose Recommendations From the Literature

After analysis of the first round, the experts will be asked to review the specific dose recommendations from the systematic literature search and select the recommendations they agree with. Additionally, they will be provided with (1) the authors’ justifications for their recommendations; (2) data from the LiverTox database; and (3) information from the corresponding SmPC on precautions, hepatotoxicity, and ADRs. If the experts completely reject the evidence, they will have the opportunity to enter their own alternative suggestions in a designated comment field. The second round is intended to allow for the analysis of differences between baseline routine clinical judgment and a subsequent evidence-informed reassessment in which experts can critically evaluate and either adopt or explicitly reject the published recommendations. Items for which first-round ratings diverge widely are of particular interest in this round as the provision of structured evidence may facilitate convergence. The design also enables meaningful analysis of substances where first-round consensus was already high to assess whether evidence confirms or challenges prevailing practice.

Of note, the provided data have the same content and structure as in the internal preassessment. For certain agents, the literature provides additional information, such as considerations for comorbidities, specific situations requiring further dose modifications, starting doses, maximum doses, or alternative agents appropriate in cirrhosis. Experts will be asked to provide a separate assessment on their agreement or disagreement for each Child-Pugh grade. The planned evaluation scales can be found in MultimediaAppendices 2  3, which provide the structured assessment forms applied in the second Delphi round. The final recommendation list will include dose recommendations for which consensus is reached.

### Application of Dose Recommendations in Patients

#### Objective of the Clinical Study and Study Design

Finally, the feasibility and acceptability of the adopted recommendations for drug selection and dosing in liver cirrhosis will be evaluated through a prospective, nonrandomized clinical study involving patients with liver cirrhosis with the following objectives: (1) improvement of drug therapy safety by applying the consensus-validated therapy recommendations in patients with liver cirrhosis and (2) demonstration of clinical usefulness of the consensus-validated therapy recommendations for patients with liver cirrhosis. For this purpose, dose recommendations will be provided to the pharmacists responsible for the wards with hepatologic patients. If discrepancies between the prescribed medication and the adopted dose recommendations are identified in a patient meeting the inclusion criteria, pharmacists will propose a dose adjustment; substitution with an alternative active substance; or another strategy mentioned in the dose recommendations, such as TDM or enhanced monitoring for adverse reactions. The acceptance of the recommendations remains at the discretion of the prescribing physician. The clinical study will be conducted in the hepatology department of University Hospital Bonn.

#### Inclusion and Exclusion Criteria

All adult (≥18 years) hospitalized patients with clinically documented liver cirrhosis of any origin and severity, except for pediatric patients, pregnant and breastfeeding women, and patients after liver transplantation, will be included. Furthermore, patients must be hospitalized for at least 24 hours and must be seen at least once by the pharmacist responsible for the ward.

#### Primary and Secondary End Points

The primary end point of the study will be the comparison of the rate of deviations from the prescribed medications before and after the implementation of the consensus-validated dose recommendations. Specifically, the rate of deviations over 9 months using the recommendations (cohort B) will be compared with the rate of deviations over 9 months before their implementation (cohort A), first identified at the prescription level and subsequently aggregated at the patient level. A deviation will be defined as any prescription of a drug covered by the recommendations that does not match the corresponding recommendation for drug selection or dose adjustment, assessed at the patient level during the hospital stay. Prescriptions are assessed at the level of individual active substances. In patients with multiple relevant prescriptions, all prescriptions are checked individually for discrepancies. For the primary end point, deviations are assessed at the patient level as a binary outcome, defined as the presence of at least one deviation during the hospital stay (yes or no). Multiple deviations per patient are aggregated, with the primary outcome defined as the presence of at least one deviation per patient. The total number of MRPs per patient, including both clinically relevant (manifest or preventable) and non–clinically relevant MRPs, will be counted and subsequently dichotomized for categorical analyses. MRPs will be identified based on the first complete medication review at initial patient assessment. Combination products will be broken down into individual active substances if these are available separately; otherwise, they will be evaluated as a single entity. Physicians are given a predefined period of 3 days to address identified MRPs. Unresolved issues will be reassessed and counted thereafter. Recurring deviations after prior resolution will be counted as new events. Deviations from recommendations are treated as a process measure of prescribing behavior, whereas secondary end points are intended to characterize feasibility, acceptance, and safety-related process signals associated with implementation of the recommendations and, therefore, are interpreted as exploratory. These may include the acceptance and rejection rates of pharmacists’ recommendations, particularly for drugs with hepatotoxic properties; frequency of typical side effects (classified as “very frequent” or “frequent” according to the SmPC) of the prescribed drugs covered by the recommendations; deviations from recommended serum levels for drugs monitored via TDM; emergency readmission rates within 30 days after discharge; and overall hospital readmission rates. Furthermore, all consensus-validated recommendations rejected by the prescribing physician will be further analyzed with regard to the corresponding reasons.

#### Sample Size Calculation and Start of Study

Currently, there is no literature available regarding inadequate dosing of drugs in hospitalized patients with liver cirrhosis. A random sample of historical data from the clinic showed an inappropriate prescribing rate of 35%. It is expected that the recommendations will reduce this rate to 20%. To detect a significant difference between the 2 cohorts using a chi-square test with the usual .05 significance level (2 sided) and 80% power, 138 patients per group are required, resulting in a total of 276 patients.

#### Data Collection and Security

The following data will be collected: age, gender, admission type (standard or emergency case), average and actual length of stay, Charlson Comorbidity Index, Child-Pugh score, type of liver cirrhosis, MRPs, severity of MRPs according to the National Coordinating Council for Medication Error Reporting and Prevention [18], ward pharmacists’ recommendations for solution of MRPs, acceptance of recommendations and reasons for rejection of an intervention by physicians, and outcome of the intervention.

The authors will only have access to pseudonymized data, whereas only the ward pharmacists and ward physicians will have access to personal data.

#### Statistical Analysis

Baseline characteristics will be summarized overall and stratified by cohort. Continuous variables will be presented as means and SDs or, in the case of skewed distributions, as medians and IQRs. Categorical variables will be reported as absolute counts and corresponding percentages. Comparisons between cohorts will be performed using appropriate statistical tests, including the *t* test (2-sided) or Wilcoxon rank-sum test for continuous variables and the chi-square test or Fisher exact test for categorical variables, as applicable. These analyses are intended to identify potential baseline imbalances between cohorts.

The primary objective is to assess whether implementation of the consensus-validated recommendations is associated with a lower rate of deviations from the recommendations regarding drug selection and dose adjustment. The primary comparison of deviations as defined in the Primary and Secondary End Points section between cohort A (before implementation) and cohort B (after implementation) will be performed using a chi-square test for the dichotomized end point; effect estimates will be reported as odds ratios with corresponding 95% CIs. Additionally, the raw number of deviations (counted as MRPs at the patient level) will be compared between the 2 cohorts using a nonparametric Wilcoxon rank-sum test. If substantial baseline differences between the cohorts are observed, adjusted analyses may be performed using regression models (eg, logistic regression for the binary outcome or Poisson or negative binomial regression for count data) including relevant baseline covariates to account for potential confounding. The main analysis will be conducted at the patient level. In a secondary analysis, the evaluation will be repeated at the prescription level; clustered data structures (multiple prescriptions per patient) will be accounted for using appropriate methods. As the primary objective is to compare prescribing behavior between 2 predefined cohorts within a real-world pretest-posttest design, the primary analysis will be conducted unadjusted for other covariates. To address potential bias, baseline characteristics will be described and compared between cohorts to assess potential imbalances. Furthermore, major organizational changes or parallel quality improvement initiatives related to medication safety will be documented and qualitatively considered when interpreting the results. An exploratory sensitivity analysis using logistic regression adjusting for key clinical variables (eg, age, sex, and Child-Pugh class) will be performed to assess robustness with regard to potential residual confounding. Secondary end points will be analyzed descriptively and, where appropriate, using inferential methods suited to the scale and distribution of the corresponding variables. If distributional assumptions for parametric testing are not met, appropriate nonparametric alternatives will be applied. Missing data will be handled via complete-case analysis without imputation; the extent and pattern of missingness will be described. No formal adjustment for multiple testing will be applied for secondary outcomes, and results will be interpreted accordingly. Analyses will be performed using R (version 4.4.2; R Foundation for Statistical Computing [19]) with RStudio (Posit PBC) [20]. Statistical significance will be set at a *P* value below .05.

### Ethical Considerations

This study is registered in the German Clinical Trials Register, a World Health Organization–recognized primary clinical trial registry under the code DRKS00033779. The protocol was approved by the Ethics Commission of the University of Bonn (2024-464-BO). As the study involves no intervention beyond routine clinical practice and only analyzes routine data from the clinical information system, patient consent is not required for participation in the study. The results will be disseminated at conference meetings and published in peer-reviewed journals.

## Results

The study was supported by funding received in January 2020 (POLAR_MI) and November 2022 (INTERPOLAR-1). The clinical validation phase of this study commenced in January 2025. As of May 2026, 121 patients have been enrolled. Statistical analyses will be conducted in accordance with the predefined analysis plan. Findings from the literature review, the Delphi consensus process, and the clinical validation phase will be reported in subsequent publications. Results of the clinical validation phase are anticipated in late August 2026.

## Discussion

During this study, we will compile evidence-based recommendations on drug selection and dose adjustments for patients with liver cirrhosis; harmonize differing dose recommendations through a Delphi consensus procedure; and apply the recommendations in hospitalized patients with liver cirrhosis in a prospective, pharmacist-led clinical intervention to analyze whether these recommendations will actually be used and contribute to patient safety. These recommendations are intended to support physicians in prescribing appropriate drugs with appropriate doses to patients with cirrhosis in clinical practice. Given the limited and sometimes heterogeneous data available for the use of drugs in liver cirrhosis, the resulting recommendations combine systematically synthesized evidence with structured expert consensus. In the absence of high-level clinical evidence (eg, randomized controlled trials), a structured Delphi consensus provides a formalized and transparent method for generating recommendations within established guideline development frameworks (eg, S2k guidelines according to Association of the Scientific Medical Societies standards) [21]. In contrast to existing drug-specific safety classifications and databases [7], this study will be the first to integrate a systematic literature search with a quality assessment to ensure the inclusion of high-quality evidence, an expert consensus process using the Delphi method, and clinical evaluation of the real-world application of the dose recommendations in hospitalized patients with cirrhosis. This clinical validation on the ward represents a key aspect of our study and differs from similar approaches such as those from Weersink et al [7,22], who developed an expert consensus–based safety classification with dose recommendations for common drugs in patients with cirrhosis. In contrast to Weersink et al [22], who directly integrated their recommendations into a clinical decision support system, our approach will follow a pharmacist-led intervention model. We will provide the developed recommendations to pharmacists working on hepatologic wards. Such investigations of pharmacist-led interventions in hospitalized patients are mainly conducted in geriatric [23-26] and, to a lesser degree, neonatal [27] and pediatric [28,29] patients. However, no such intervention has been conducted specifically in patients with cirrhosis so far. As in similar studies, we will analyze MRPs such as patient adherence [30] and occurrence of ADRs [31] using the dose recommendations identified during the first part of our study.

As an additional unique feature of our approach, the second Delphi round represents a structured re-evaluation under enriched information conditions rather than a purely iterative consensus process. In this context, we plan to specifically provide information about the hepatotoxic effects of the drugs to the experts during the consensus process whenever such data are available [32]. Although Zimmerman [33] reported in 1978 that patients with cirrhosis do not experience drug-induced hepatotoxic effects more frequently than other patients, such events are associated with higher morbidity and mortality due to the preexisting vulnerability. Although only a few publications consider hepatotoxicity in their dose recommendations, we want to analyze whether this information from the literature and the SmPC affects expert decisions regarding dose adjustments. Our modified design of the 2-round consensus procedure allows for the analysis of how clinicians actually manage drug dosing in patients with cirrhosis in the absence of formal guidance and the comparison of how the provision of structured evidence influences expert decisions—a dimension not captured through the conventional approach.

One limitation of our study is the evaluation of a restricted number of drugs. However, we will focus on the drugs with the highest expected clinical relevance for the treatment of patients with liver cirrhosis, determined via reconciliation with the stock list of a hospital of maximum care covering all clinically common drugs and the assessment of relevance based on frequency of use, and on the need for dose adjustments in patients with cirrhosis, determined via an internal consensus procedure. In the future, this initial evaluation could be expanded to additional active substances following the same methodology. The weighting of assessment criteria and the predefined cutoff value for inclusion represent pragmatic thresholds defined to operationalize this prioritization strategy. These parameters are not based on statistical calculations and may limit external validity. As prescribing patterns, formularies, and patient populations may vary across institutions, the prioritization strategy applied in this study may reflect local clinical practice and, therefore, may require contextual adaptation when transferred to other health care settings.

In addition, the Child-Pugh classification [15,16] was used as the primary framework for drug selection and dose adjustment for this study. While the Child-Pugh classification reflects routine clinical practice and forms the basis of most available dosing guidance, it can only serve as a surrogate for hepatic drug metabolism and does not capture other relevant determinants of pharmacokinetics such as renal dysfunction, systemic inflammation, sarcopenia, or portosystemic shunting. Furthermore, the Delphi process is based on the simplified general model of an average hospitalized patient with cirrhosis, excluding atypical or non–cirrhosis-related comorbidities that may independently influence dosing decisions. Accordingly, the resulting recommendations should be interpreted as a basic framework requiring individualized clinical judgment in this heterogeneous patient population. A further limitation concerns the design of the clinical validation phase, which aims to analyze the feasibility and acceptability of the dose recommendations in clinical practice. The pretest-posttest, nonrandomized evaluation reflects real-world constraints but does not allow for causal inference regarding the effectiveness of the recommendations. Observed changes in prescribing behavior may be influenced by temporal effects, increased awareness, or concurrent changes in clinical practice. However, efforts will be made to mitigate these effects by maintaining a stable clinical setting across both study periods (before and after implementation of the recommendations), with consistent senior and ward physicians, and by prospectively capturing physician responses to pharmacist interventions. Both cohorts will be assessed consecutively without temporal gaps and cover comparable time frames, reducing the likelihood of structural changes in care processes or systematic differences in patient management. A prior literature review did not indicate relevant seasonal variation in disease severity of liver cirrhosis that would be expected to systematically bias the comparison. In addition, the primary end point focuses on deviations from recommendations as a process measure rather than clinical outcomes. Further clinical studies with different designs, alternative end points, and larger patient cohorts will be needed to investigate clinical outcomes on a broader scale.

In conclusion, this protocol describes a structured method to establish clinical guidance by compiling and standardizing dose recommendations for patients with liver cirrhosis followed by clinical validation. This approach has the potential to enhance the safety of patients with cirrhosis and contribute to better clinical outcomes.

## Supplementary material

10.2196/89042 Multimedia Appendix 1 Preassessment scoring sheet for clinical relevance, frequency of prescription, and expected benefit.

10.2196/89042 Multimedia Appendix 2 Assessment sheet for Delphi round 1 (usual prescribing).

10.2196/89042 Multimedia Appendix 3 Assessment sheet for Delphi round 2 (dose recommendation selection).

10.2196/89042 Checklist 1 PRISMA, CREDES, and SPIRIT checklists.

## Abbreviations

ADR: adverse drug reaction
AGREE: Appraisal of Guidelines for Research and Evaluation
CREDES: Guidance on Conducting and Reporting Delphi Studies
MRP: medication-related problem
PRISMA: Preferred Reporting Items for Systematic Reviews and Meta-Analyses
SmPC: Summary of Product Characteristics
SPIRIT: Standard Protocol Items: Recommendations for Interventional Trials
TDM: therapeutic drug monitoring

## References

[1] Gonzales HM, Fleming JN, Gebregziabher M (2021). Pharmacist-led mobile health intervention and transplant medication safety: a randomized controlled clinical trial. Clin J Am Soc Nephrol.

[2] Verbeeck RK (2008). Pharmacokinetics and dosage adjustment in patients with hepatic dysfunction. Eur J Clin Pharmacol.

[3] Krähenbühl-Melcher A, Schlienger R, Lampert M, Haschke M, Drewe J, Krähenbühl S (2007). Drug-related problems in hospitals: a review of the recent literature. Drug Saf.

[4] Schuppan D, Afdhal NH (2008). Liver cirrhosis. Lancet.

[5] Franz CC, Hildbrand C, Born C, Egger S, Rätz Bravo AE, Krähenbühl S (2013). Dose adjustment in patients with liver cirrhosis: impact on adverse drug reactions and hospitalizations. Eur J Clin Pharmacol.

[6] Cheema E, Al-Aryan A, Al-Hamid A (2019). Medicine use and medicine-related problems in patients with liver cirrhosis: a systematic review of quantitative and qualitative studies. Eur J Clin Pharmacol.

[7] Weersink RA, Bouma M, Burger DM (2018). Evidence-based recommendations to improve the safe use of drugs in patients with liver cirrhosis. Drug Saf.

[8] Westphal JF, Brogard JM (1997). Drug administration in chronic liver disease. Drug Saf.

[9] Weisbach L, Schuster AK, Hartmann M, Fromm MF, Maas R, Farker K (2022). Inconsistencies and ambiguities in liver-disease-related contraindications-a systematic analysis of SmPCs/PI of major drug markets. J Clin Med.

[10] Karsten Dafonte K, Weber L, Chmielewski F (2023). Dose recommendations for common drugs in patients with liver cirrhosis: a systematic literature review. Clin Drug Investig.

[11] OCEBM levels of evidence. Centre for Evidence-Based Medicine.

[12] Page MJ, McKenzie JE, Bossuyt PM (2021). The PRISMA 2020 statement: an updated guideline for reporting systematic reviews. BMJ.

[13] Brouwers MC, Kerkvliet K, Spithoff K, AGREE Next Steps Consortium (2016). The AGREE Reporting Checklist: a tool to improve reporting of clinical practice guidelines. BMJ.

[14] Jünger S, Payne SA, Brine J, Radbruch L, Brearley SG (2017). Guidance on Conducting and REporting DElphi Studies (CREDES) in palliative care: recommendations based on a methodological systematic review. Palliat Med.

[15] Child CG, Turcotte JG (1964). Surgery and portal hypertension. Major Probl Clin Surg.

[16] Pugh RN, Murray-Lyon IM, Dawson JL, Pietroni MC, Williams R (1973). Transection of the oesophagus for bleeding oesophageal varices. Br J Surg.

[17] Niederberger M, Spranger J (2020). Delphi technique in health sciences: a map. Front Public Health.

[18] National Coordinating Council for Medication Error Reporting and Prevention.

[19] R Core Team (2021). R: a language and environment for statistical computing. R Foundation for Statistical Computing.

[20] (2024). RStudio: integrated development environment for R. Posit Software.

[21] (2023). AWMF guidance manual and rules for guideline development. https://www.awmf.org/fileadmin/user_upload/dateien/downloads_regelwerk/awmf-regelwerk-en-2023-v2.1.pdf.

[22] Weersink RA, Bouma M, Burger DM (2016). Evaluating the safety and dosing of drugs in patients with liver cirrhosis by literature review and expert opinion. BMJ Open.

[23] Lee S, Yu YM, Han E, Park MS, Lee JH, Chang MJ (2023). Effect of pharmacist-led intervention in elderly patients through a comprehensive medication reconciliation: a randomized clinical trial. Yonsei Med J.

[24] van Nuland M, Butterhoff M, Verwijmeren K (2023). Assessment of drug-related problems at the emergency department in older patients living with frailty: pharmacist-led medication reviews within a geriatric care team. BMC Geriatr.

[25] Ravn-Nielsen LV, Duckert ML, Lund ML (2018). Effect of an in-hospital multifaceted clinical pharmacist intervention on the risk of readmission: a randomized clinical trial. JAMA Intern Med.

[26] Gustafsson M, Sjölander M, Pfister B, Jonsson J, Schneede J, Lövheim H (2017). Pharmacist participation in hospital ward teams and hospital readmission rates among people with dementia: a randomized controlled trial. Eur J Clin Pharmacol.

[27] Yalçın N, Kaşıkcı M, Çelik HT, Allegaert K, Demirkan K, Yiğit Ş (2023). Impact of clinical pharmacist-led intervention for drug-related problems in neonatal intensive care unit a randomized controlled trial. Front Pharmacol.

[28] Orth LE, Feudtner C, Kempe A (2023). A coordinated approach for managing polypharmacy among children with medical complexity: rationale and design of the Pediatric Medication Therapy Management (pMTM) randomized controlled trial. BMC Health Serv Res.

[29] Jarad S, Akour A, Khreisat WH, Elshammari AK, Madae’en S (2022). The role of clinical pharmacist in pediatrics’ adherence to antiepileptic drugs. J Pharm Technol.

[30] Wu M, Xu X, Zhao R, Bai X, Zhu B, Zhao Z (2023). Effect of pharmacist-led interventions on medication adherence and glycemic control in type 2 diabetic patients: a study from the Chinese population. Patient Prefer Adherence.

[31] Brajković A, Bićanić LA, Strgačić M, Orehovački H, Ramalho-de-Oliveira D, Mucalo I (2022). The impact of pharmacist-led medication management services on the quality of life and adverse drug reaction occurrence. Pharmacy (Basel).

[32] Schlatter C, Egger SS, Tchambaz L, Krähenbühl S (2009). Pharmacokinetic changes of psychotropic drugs in patients with liver disease: implications for dose adaptation. Drug Saf.

[33] Zimmerman HJ (1978). Drug-induced liver disease. Drugs.

