# Supplementary material for: Dose Recommendations for Drugs in Patients With Liver Cirrhosis (The ALIVe Study): Protocol for a Multiphase Validation and Consensus Study
Source: JMIR Res Protoc. 2026 Jun 9;15:e89042. doi: 10.2196/89042 (PMC13249594; doi:10.2196/89042)
Supplement: Multimedia Appendix 1 [file resprot-v15-e89042-s001.pdf]

Additional file 1: Evaluation scales for the internal pre-assessment

Evaluation scales		
**Relevance of the Adverse Events**	**Frequency of Application**	**Expected Benefit of a Dose Recommendation**
Please imagine an average hospital patient with liver cirrhosis during his hospital stay....		
In your opinion, how important is it, in terms of patient safety, to **avoid the side effects** listed below, considering their frequency in patients with liver cirrhosis?	In your opinion, how important are clear dosage recommendations based on **frequency of prescription**, especially in patients with liver cirrhosis?	In your opinion, how **useful are clear dose recommendations** for active substances in order to prevent both general side effects and those specifically listed below in patients with cirrhosis of the liver?
0 = No Evaluation Possible (please clarify)	0 = No Evaluation Possible (please clarify)	0 = No Evaluation Possible (please clarify)
1 = No / Negligible Priority	1 = No / Negligible Priority	1 = Not Useful
2 = Medium Priority	2 = Medium Priority	2 = Useful
3 = High Priority	3 = High Priority	3 = Very Useful/Recommended
