# Supplementary material for: Dose Recommendations for Drugs in Patients With Liver Cirrhosis (The ALIVe Study): Protocol for a Multiphase Validation and Consensus Study
Source: JMIR Res Protoc. 2026 Jun 9;15:e89042. doi: 10.2196/89042 (PMC13249594; doi:10.2196/89042)
Supplement: Multimedia Appendix 2 [file resprot-v15-e89042-s002.pdf]

Additional file 2: Evaluation scales for Delphi assessment, Round 1

Active substance (sorted by ATC code)	Rating scales					
	**How do you handle the following agents in routine clinical practice?**					
	I agree with the following statement... Please tick only one statement for each individual Child-Pugh stage and refer to an **average hospitalised** patient with liver cirrhosis!					
	**Child Pugh A**		**Child Pugh B**		**Child Pugh C**	
**Metoclopramide**	I do not make any dose adjustment --> Administration of normal dose	☐	I do not make any dose adjustment --> Administration of normal dose	☐	I do not make any dose adjustment --> Administration of normal dose	☐
	I avoid the active substance in liver cirrhosis	☐	I avoid the active substance in liver cirrhosis	☐	I avoid the active substance in liver cirrhosis	☐
	I adjust the dose	☐	I adjust the dose	☐	I adjust the dose	☐
	--> In case of dose adjustment, please explain on the right!					

Rating scales			
**Only (!) in case of a dose adjustment**			
I agree with the following statement... **You can tick several boxes here.** Please decide on **at least one statement** only for that **Child-Pugh stage** for which you are **making a dose adjustment**!			
	**Child Pugh A**	**Child Pugh B**	**Child Pugh C**
I reduce the dose *(please explain under "comments" by how much [unit])*	☐	☐	☐
I extend the dosing interval *(please explain under "comments" by how many hours)*	☐	☐	☐
I do something else (*please explain under "comments"*)	☐	☐	☐

Comments:
