# Supplementary material for: Dose Recommendations for Drugs in Patients With Liver Cirrhosis (The ALIVe Study): Protocol for a Multiphase Validation and Consensus Study
Source: JMIR Res Protoc. 2026 Jun 9;15:e89042. doi: 10.2196/89042 (PMC13249594; doi:10.2196/89042)
Supplement: Multimedia Appendix 3 [file resprot-v15-e89042-s003.pdf]

**Additional file 3:** Evaluation scales for Delphi assessment, Round 2

| Active substance (sorted by ATC code) | Majority decision from round 1 | Your personal ratings of round 1 | | (Safety) information especially for patients with liver cirrhosis | Frequency of the side effect (especially in liver cirrhosis - if known) |
|---|---|---|---|---|---|
| **Metoclopramide**<br>(Route of administration: peroral, tablets)<br>(Indication: Nausea and Vomiting) | **Child Pugh A**<br>*Example:*<br>No dose adjustment required<br>Consensus (XX) | **Child Pugh A**<br>*Example:*<br>No dose adjustment required | Information from the Literature | - Increased sleepiness in liver cirrhotics [2] | Not specified/Not known/Conflicting data |
| | | | | - Increased fluid retention in liver cirrhotics [2,4] | Not specified/Not known/Conflicting data |
| | | | | - Occurence of extrapyrimidal side effects in liver cirrhosis (high dose and several days of use) [2] | Individual cases |
| | **Child Pugh B**<br>*Example:*<br>No dose adjustment required<br>Majority approval (XX) | **Child Pugh B**<br>*Example:*<br>No dose adjustment required | Information from the SmPC | Dosage: "In patients with severe hepatic impairment, decrease the dosage by 50%" | |
| | **Child Pugh C**<br>*Example:*<br>Dose adjustment required<br>Majority approval (XX) | **Child Pugh C**<br>*Example:*<br>Dose adjustment required<br>(Reduction by 50%) | Information from the LiverTox database | LiverTox Likelihood score: C (probable cause of clinically apparent liver injury) | |
| | *Comments:* | | | | |

**Evaluation**

**Evaluation on specific dose adjustments**

***I agree with the following statement...***
(please decide on a statement **for each Child-Pugh stage**!
Please check the evidence provided (safety information as well as suggested dose modifications for **maintenance dosages**)).

| Child Pugh A | | Child Pugh B | | Child Pugh C | |
|---|---|---|---|---|---|
| No dose adjustment necessary --> administration as in liver-healthy patients [8] | ☐ | No dose adjustment necessary --> administration as in liver-healthy patients [8] | ☐ | No dose adjustment necessary --> administration as in liver-healthy patients [8] | ☐ |
| Reduction of the dose by 50% compared to the standard dose in liver-healthy people [4,16] | ☐ | Reduction of the dose by 50% compared to the standard dose in liver-healthy people [4,16] | ☐ | Reduction of the dose by 50% compared to the standard dose in liver-healthy people [4,16, SmPC] | ☐ |
| XXX<br>[Expert opinion] | ☐ | XXX<br>[Expert opinion] | ☐ | XXX<br>[Expert opinion] | ☐ |
| I do not agree with the recommendations above *(please indicate reason and alternative course of action under "Comments" !)* | ☐ | I do not agree with the recommendations above (please indicate reason and alternative course of action under *"Comments"* !) | ☐ | I do not agree with the recommendations above (please indicate reason and alternative course of action under *"Comments"* !) | ☐ |
| I cannot give a rating - **only in exceptional cases!** *(please indicate reason under "Comments" !)* | ☐ | I cannot give a rating - **only in exceptional cases!** *(please indicate reason under "Comments" !)* | ☐ | I cannot give a rating - **only in exceptional cases!** *(please indicate reason under "Comments" !)* | ☐ |

Metoclopramide illustrates the structure of the evaluation scale as an example.

*Abbrevations:*

XX: Determined approval in [%].

XXX: Placeholder for expert opinions from the 1st round, which will be provided for evaluation in the 2nd round.
