# Supplementary material for: Dose Recommendations for Drugs in Patients With Liver Cirrhosis (The ALIVe Study): Protocol for a Multiphase Validation and Consensus Study
Source: JMIR Res Protoc. 2026 Jun 9;15:e89042. doi: 10.2196/89042 (PMC13249594; doi:10.2196/89042)
Supplement: Checklist 1 [file resprot-v15-e89042-s004.pdf]

Additional File 1a. PRISMA 2020 checklist (adapted for a study protocol integrating a previously published systematic review [10])

Section and Topic	Item #	Checklist item	Location where item is reported	Status and Justification
TITLE	TITLE	TITLE		
Title	1	Identify the report as a systematic review.	Title: “The ALIVe Study: A Study Protocol for the Compilation of Literature…”	Partially addressed. The manuscript is clearly identified as a study protocol but not explicitly labeled as a standalone systematic review in the title. The literature phase is integrated into a multi-step methodological framework rather than presented as an independent review publication.
ABSTRACT	ABSTRACT	ABSTRACT		
Abstract	2	See the PRISMA 2020 for Abstracts checklist.	Not applicable.	Not applicable. This manuscript is a study protocol and does not report completed systematic review results; therefore, a PRISMA-conform abstract is not required.
INTRODUCTIO N	INTRODUCTIO N	INTRODUCTION		
Rationale	3	Describe the rationale for the review in the context of existing knowledge.	Introduction, beginning “Liver cirrhosis with advanced fibrotic remodelling…”	Addressed. The rationale for compiling structured dosing recommendatio

				ns is clearly described in the context of existing literature and identified evidence gaps.
Objectives	4	Provide an explicit statement of the objective(s) or question(s) the review addresses.	Introduction, paragraph beginning “The objective of this study is to provide guidance…”	Addressed. The objectives of the literature compilation and harmonization process are explicitly stated.
METHODS	METHODS	METHODS		
Eligibility criteria	5	Specify the inclusion and exclusion criteria for the review and how studies were grouped for the syntheses.	Section “Structured Literature Search and Selection of Literature…”	Partially addressed. Inclusion criteria are described; detailed criteria are reported in the previously published systematic review [10].
Information sources	6	Specify all databases, registers, websites, organisations, reference lists and other sources searched or consulted to identify studies. Specify the date when each source was last searched or consulted.	Section “Structured searches of PubMed and Embase were conducted…”	Addressed. All databases used for identification of relevant literature are explicitly stated.
Search strategy	7	Present the full search strategies for all databases, registers and websites, including any filters and limits used.	Section referring to detailed search strategy reported in original publication [10].	Partially addressed. Full search strategies are not reproduced here but are fully available in the previously published review.

Selection process	8	Specify the methods used to decide whether a study met the inclusion criteria of the review, including how many reviewers screened each record and each report retrieved, whether they worked independently, and if applicable, details of automation tools used in the process.	Section "Structured Literature Search and Selection of Literature…"	Partially addressed. The general selection process is described; detailed screening procedures are reported in the prior review.
Data collection process	9	Specify the methods used to collect data from reports, including how many reviewers collected data from each report, whether they worked independently, any processes for obtaining or confirming data from study investigators, and if applicable, details of automation tools used in the process.	Section "Extraction of Information for Each Active Substance…"	Partially addressed. Data extraction is described; reviewer independence details are reported in the prior publication.
Data items	10a	List and define all outcomes for which data were sought. Specify whether all results that were compatible with each outcome domain in each study were sought	Section defining explicit and clinically applicable dose recommendatio ns.	Addressed. Explicit, clinically applicable dose recommendatio ns stratified by Child–Pugh class are clearly defined.

		(e.g. for all measures, time points, analyses), and if not, the methods used to decide which results to collect.		
Data items	10b	List and define all other variables for which data were sought (e.g. participant and intervention characteristics, funding sources). Describe any assumptions made about any missing or unclear information.	Section "Assessment of Reporting Quality and Evidence Level…"	Addressed. Additional variables including reporting quality and evidence levels (CEBM) are operationalized.
Study risk of bias assessment	11	Specify the methods used to assess risk of bias in the included studies, including details of the tool(s) used, how many reviewers assessed each study and whether they worked independently, and if applicable, details of automation tools used in the process.	Section "Assessment of Reporting Quality and Evidence Level…"	Partially addressed. No classical risk-of-bias tool for RCTs was applied; reporting quality and evidence levels were assessed instead.
Effect measures	12	Specify for each outcome the effect measure(s) (e.g. risk ratio, mean difference) used in the synthesis or presentation of results.	Not applicable.	Not applicable. No quantitative synthesis or meta-analysis was performed.
Synthesis methods	13a	Describe the processes used to decide which studies were	Not applicable.	Not applicable. No quantitative synthesis or meta-analysis

		eligible for each synthesis (e.g. tabulating the study intervention characteristics and comparing against the planned groups for each synthesis (item #5)).		was performed.
Synthesis methods	13b	Describe any methods required to prepare the data for presentation or synthesis, such as handling of missing summary statistics, or data conversions.	Not applicable.	Not applicable. No quantitative synthesis or meta-analysis was performed.
Synthesis methods	13c	Describe any methods used to tabulate or visually display results of individual studies and syntheses.	Not applicable.	Not applicable. No quantitative synthesis or meta-analysis was performed.
Synthesis methods	13d	Describe any methods used to synthesize results and provide a rationale for the choice(s). If meta-analysis was performed, describe the model(s), method(s) to identify the presence and extent of statistical heterogeneity, and software package(s) used.	Not applicable.	Not applicable. No quantitative synthesis or meta-analysis was performed.
Synthesis methods	13e	Describe any methods used to explore possible causes of	Not applicable.	Not applicable. No quantitative synthesis or meta-analysis

		heterogeneity among study results (e.g. subgroup analysis, meta-regression).		was performed.
Synthesis methods	13f	Describe any sensitivity analyses conducted to assess robustness of the synthesized results.	Not applicable.	Not applicable. No quantitative synthesis or meta-analysis was performed.
Reporting bias assessment	14	Describe any methods used to assess risk of bias due to missing results in a synthesis (arising from reporting biases).	Not applicable.	Not applicable. No reporting bias assessment required due to absence of quantitative synthesis.
Certainty assessment	15	Describe any methods used to assess certainty (or confidence) in the body of evidence for an outcome.	Section "Evidence levels were assigned according to the Oxford Centre for Evidence-Based Medicine…"	Addressed (modified). Certainty was operationalized using CEBM evidence levels rather than GRADE.
RESULTS	RESULTS	RESULTS		
Study selection	16a	Describe the results of the search and selection process, from the number of records identified in the search to the number of studies included in the review, ideally using a flow diagram.	Results of systematic review published separately in [10].	Not applicable in this protocol. This manuscript builds upon a previously published systematic review.
Study selection	16b	Cite studies that might appear to meet the inclusion criteria, but which were excluded, and explain why	Results of systematic review published separately in [10].	Not applicable in this protocol. This manuscript builds upon a previously published

		they were excluded.		systematic review.
Study characteristics	17	Cite each included study and present its characteristics.	Results of systematic review published separately in [10].	Not applicable in this protocol. This manuscript builds upon a previously published systematic review.
Risk of bias in studies	18	Present assessments of risk of bias for each included study.	Results of systematic review published separately in [10].	Not applicable in this protocol. This manuscript builds upon a previously published systematic review.
Results of individual studies	19	For all outcomes, present, for each study: (a) summary statistics for each group (where appropriate) and (b) an effect estimate and its precision (e.g. confidence/credible interval), ideally using structured tables or plots.	Results of systematic review published separately in [10].	Not applicable in this protocol. This manuscript builds upon a previously published systematic review.
Results of syntheses	20a	For each synthesis, briefly summarise the characteristics and risk of bias among contributing studies.	Not applicable.	Not applicable. No quantitative synthesis or meta-analysis was performed.
Results of syntheses	20b	Present results of all statistical syntheses conducted. If meta-analysis was done, present for each the summary estimate and its	Not applicable.	Not applicable. No quantitative synthesis or meta-analysis was performed.

		precision (e.g. confidence/credible interval) and measures of statistical heterogeneity. If comparing groups, describe the direction of the effect.		
Results of syntheses	20c	Present results of all investigations of possible causes of heterogeneity among study results.	Not applicable.	Not applicable. No quantitative synthesis or meta-analysis was performed.
Results of syntheses	20d	Present results of all sensitivity analyses conducted to assess the robustness of the synthesized results.	Not applicable.	Not applicable. No quantitative synthesis or meta-analysis was performed.
Reporting biases	21	Present assessments of risk of bias due to missing results (arising from reporting biases) for each synthesis assessed.	Not applicable.	Not applicable. No quantitative synthesis or meta-analysis was performed.
Certainty of evidence	22	Present assessments of certainty (or confidence) in the body of evidence for each outcome assessed.	Results of systematic review published separately in [10].	Not applicable in this protocol. This manuscript builds upon a previously published systematic review.
DISCUSSION	DISCUSSION	DISCUSSION		
Discussion	23a	Provide a general interpretation of the results in the context of other evidence.	Discussion, beginning "During the ALIVe-study we will compile…"	Partially addressed. Discussion reflects methodological implications but does not interpret review

				results, as this is a protocol publication.
Discussion	23b	Discuss any limitations of the evidence included in the review.	Discussion, paragraph beginning “One limitation of our study is…”	Partially addressed. General limitations of the evidence base are discussed. Detailed appraisal of included studies is reported in the previously published systematic review [10], on which this protocol builds.
Discussion	23c	Discuss any limitations of the review processes used.	Discussion, paragraph addressing methodological limitations of the structured literature compilation.	Addressed . Methodological limitations of the literature compilation (heterogeneous sources, reliance on published recommendatio ns, Child–Pugh framework) are discussed. No quantitative synthesis was performed.
Discussion	23d	Discuss implications of the results for practice, policy, and future research.	Discussion, concluding paragraph beginning “In conclusion, this protocol describes…”	Addressed. Implications for clinical practice and the need for prospective validation and implementation studies are outlined as subsequent phases of the ALIVe study.
OTHER INFORMATIO N	OTHER INFORMATIO N	OTHER INFORMATION		

Registration and protocol	24a	Provide registration information for the review, including register name and registration number, or state that the review was not registered.	Section "Trial registration" and "Ethics Approval and Consent to Participate…"	Addressed. The clinical phase is registered; the literature review was not prospectively registered and this is transparently stated.
Registration and protocol	24b	Indicate where the review protocol can be accessed, or state that a protocol was not prepared.	Section "Trial registration" and statement "This manuscript constitutes the study protocol…"	Addressed. This manuscript constitutes the published study protocol. The underlying systematic review was not prospectively registered.
Registration and protocol	24c	Describe and explain any amendments to information provided at registration or in the protocol.	Not applicable	No amendments to a registered review protocol were made. The current manuscript represents the initial protocol publication (Version 1.0).
Support	25	Describe sources of financial or non-financial support for the review, and the role of the funders or sponsors in the review.	Section "Funding."	Addressed. Funding sources are declared.
Competing interests	26	Declare any competing interests of review authors.	Section "Competing interests."	Addressed. Competing interests are declared.
Availability of data, code and other materials	27	Report which of the following are publicly available and where they can be found: template data	Section "Availability of data and materials."	Addressed. Data availability statement provided.

		collection forms; data extracted from included studies; data used for all analyses; analytic code; any other materials used in the review.		

Additional File 1b. CREDES reporting checklist (adapted for Delphi Rounds 1 and 2 of the ALIVe study; documenting the methodological framework of the consensus process only)

CREDES Domain	Domain Description	Location where item is reported	Status and Justification
I. TITLE AND ABSTRACT	Identification of the study as a Delphi process in the title and abstract, and provision of a structured summary.	Title and Abstract (Delphi explicitly described in title and methods section).	Addressed. The study is clearly identified as a Delphi-based process and the abstract provides a structured summary of objectives, methods, and results.
II. EPISTEMOLOGY	Positioning within a strand of theory of science (e.g., realist or constructivist), including statement of objectives and preliminary assumptions.	Methods section describing predefined quantitative consensus thresholds and statistical analysis.	Partially addressed. An explicit epistemological positioning is not formally stated; however, the study follows a quantitative consensus framework operationalised through predefined agreement thresholds and statistical testing.
III. FORMAL CONTEXT	Information on the research team, project background, funding, study protocol, and ethics approval.	Author affiliations; sections 'Funding', 'Ethics Approval and Consent to Participate', and trial registration statement.	Addressed. Research team composition, funding sources, ethical approval, and protocol registration are transparently reported.
IV. KNOWLEDGE BASE	Description of the evidence base and how current state of research is considered or integrated.	Introduction and section describing literature summaries, SmPC excerpts, and LiverTox integration.	Addressed. The Delphi process is explicitly based on a structured evidence base including a systematic review, regulatory information, and

			hepatotoxicity databases.
V. KNOWLEDGE AND KNOWLEDGE INTEGRATION	Specification of relevant knowledge stocks and perspectives, and how they are integrated.	Section 'Expert panel and study design' and description of interdisciplinary composition and feedback process.	Addressed. Complementary expertise (hepatology, clinical pharmacology, pharmacy) is intentionally integrated and harmonised through structured feedback between rounds.
VI. DELPHI VARIANT AND ROLE IN RESEARCH PROCESS	Statement and justification of chosen Delphi variant and its role within the research process.	Methods beginning 'This second round of a two-step Delphi-based study…'	Addressed. A modified two-round Delphi design is clearly described and situated within the broader ALIVe multi-phase research framework.
VII. SAMPLE	Comprehensive description of expert panel, recruitment, expertise, number per round, handling of anonymity and dropouts.	Section 'Expert panel and study design' and Results reporting 100% response rate.	Addressed. Panel size, disciplines, expertise criteria, anonymity of voting, and absence of attrition (100% response rate) are reported.
VIII. SURVEY INSTRUMENT	Description of scope, structure, derivation and testing of questions, scale types, and design of survey instrument.	Section 'Questionnaire and provided information…'	Partially addressed. Questionnaire structure and predefined response options are described; formal pilot testing is not separately reported.
IX. DELPHI ROUNDS	Number of Delphi rounds and disclosure of termination criterion.	Methods describing Round 1 and Round 2 structure and predefined stopping after Round 2.	Addressed. Two predefined rounds were conducted and stopping criteria were clearly defined.
X. FEEDBACK	Disclosure of statistical and graphic	Methods describing provision of individual and	Addressed. Structured statistical feedback,

	representation of feedback per round.	aggregated Round 1 responses in Round 2.	including prior individual responses and group consensus levels, was provided between rounds.
XI. EVALUATION	Definition and handling of consensus, quantitative and qualitative evaluation strategies, and how they are combined.	Section 'Consensus levels…' and statistical analysis section (McNemar's test; regression models).	Addressed.<br>Explicit consensus thresholds were predefined and quantitative evaluation strategies were clearly described.
XII. RESULT	Presentation of the Delphi process (response, procedure, modifications) and results of individual rounds.	Results section reporting consensus shifts, response rates, and round comparisons.	Addressed.<br>Response rates, consensus levels, and changes between rounds are transparently reported; modifications between rounds are explained.
XIII. QUALITY OF DATA AND INTERPRETATION	Reflection on quality of data collection and evaluation process, including quality criteria.	Discussion section addressing strengths and methodological limitations of the Delphi process.	Partially addressed.<br>Strengths and limitations of the Delphi methodology are discussed; formal quality criteria beyond consensus thresholds are not separately defined.
XIV. DISCUSSION AND LIMITATIONS OF THE FINDINGS	Critical reflection on validity of findings and limitations.	Discussion section addressing generalisability and methodological limitations.	Addressed.<br>Limitations of the consensus findings and issues of generalisability are critically discussed.
XV. DISSEMINATION	Statement on how findings will be processed or used beyond the Delphi study.	Discussion concluding section describing pilot implementation and planned validation phases.	Addressed.<br>The intended clinical application of the consensus recommendations and planned prospective validation studies

			are clearly described.

Additional File 1c. SPIRIT 2025 checklist of items to address in a randomized trial protocol*

Section / Topic	No	SPIRIT 2025 checklist item description	Reported on page no.
**Administrative information**			
Title and structured summary	1a	Title stating the trial design, population, and interventions, with identification as a protocol	Addressed. Title page: Title specifies protocol, population and intervention
	1b	Structured summary of trial design and methods, including items from the World Health Organization Trial Registration Data Set	Addressed. Abstract: Structured summary provided
Protocol version	2	Version date and identifier	Addressed. Title page: Version reflected in manuscript versioning
Roles and responsibilities	3a	Names, affiliations, and roles of protocol contributors	Addressed. Author contributions
	3b	Name and contact information for the trial sponsor	Addressed. Funding / Declarations
	3c	Role of trial sponsor and funders in design, conduct, analysis, and reporting of trial; including any authority over these activities	Partially addressed. Declarations: Funding stated, influence not fully detailed
	3d	Composition, roles, and responsibilities of the coordinating site, steering committee, endpoint adjudication committee, data management team, and other individuals or groups overseeing the trial, if applicable	Not applicable. Not described: No trial oversight committees
**Open science**			
Trial registration	4	Name of trial registry, identifying number (with URL), and date of registration. If not yet registered, name of intended registry	Addressed. Methods / Abstract: DRKS registration provided
Protocol and statistical analysis plan	5	Where the trial protocol and statistical analysis plan can be accessed	Partially addressed. Manuscript: Protocol described but no explicit access statement
Data sharing	6	Where and how the individual de-identified participant data (including data dictionary), statistical code, and any other materials will be accessible	Addressed. Declarations: Data sharing described
Funding and conflicts of interest	7a	Sources of funding and other support (e.g., supply of drugs)	Addressed. Funding
	7b	Financial and other conflicts of interest for principal investigators and steering committee members	Addressed. Declarations

Dissemination policy	8	Plans to communicate trial results to participants, healthcare professionals, the public, and other relevant groups (e.g., reporting in trial registry, plain language summary, publication)	Addressed. Ethics / Dissemination: Publication and dissemination plans described.
**Introduction**			
Background and rationale	9a	Scientific background and rationale, including summary of relevant studies (published and unpublished) examining benefits and harms for each intervention	Addressed. Introduction
	9b	Explanation for choice of comparator	Partially addressed. Introduction: Comparator implicit in pre-post design but not explicitly framed.
Objectives	10	Specific objectives related to benefits and harms	Addressed. Introduction / Methods
**Methods: Patient and public involvement, trial design**			
Patient and public involvement	11	Details of, or plans for, patient or public involvement in the design, conduct, and reporting of the trial	Not applicable. Not described: No patient involvement
Trial design	12	Description of trial design including type of trial (e.g., parallel group, crossover), allocation ratio, and framework (e.g., superiority, equivalence, non-inferiority, exploratory)	Addressed. Methods: Pre-post cohort design described
**Methods: Participants, interventions, and outcomes**			
Trial setting	13	Settings (e.g., community, hospital) and locations (e.g., countries, sites) where the trial will be conducted	Addressed. Methods – Setting
Eligibility criteria	14 a	Eligibility criteria for participants	Addressed. Methods – Eligibility criteria
	14 b	If applicable, eligibility criteria for sites and for individuals who will deliver the interventions (e.g., surgeons, physiotherapists)	Partially addressed. Methods: Study setting described, no formal site eligibility criteria.
Intervention and comparator	15 a	Intervention and comparator with sufficient details to allow replication including how, when, and by whom they will be administered. If relevant, where additional materials describing the intervention and comparator (e.g., intervention manual) can be accessed	Addressed. Methods – Intervention
	15 b	Criteria for discontinuing or modifying allocated intervention/comparator for a trial participant (e.g., drug dose change in response to harms, participant request, or improving/worsening disease)	Not applicable. Routine care: No intervention beyond routine care
	15 c	Strategies to improve adherence to intervention/comparator protocols, if applicable,	Not applicable. Routine care

		and any procedures for monitoring adherence (e.g., drug tablet return, sessions attended)	
	15 d	Concomitant care that is permitted or prohibited during the trial	Addressed. Methods: Routine care context defines concomitant care
Outcomes	16	Primary and secondary outcomes, including the specific measurement variable (e.g., systolic blood pressure), analysis metric (e.g., change from baseline, final value, time to event), method of aggregation (e.g., median, proportion), and time point for each outcome	Addressed. Methods – Outcomes / Statistical analysis
Harms	17	How harms are defined and will be assessed (e.g., systematically, non-systematically)	Partially addressed. Methods: Harms indirectly reflected via MRPs
Participant timeline	18	Time schedule of enrollment, interventions (including any run-ins and washouts), assessments, and visits for participants. A schematic diagram is highly recommended (see Figure)	Partially addressed. Methods: No schematic timeline
Sample size	19	How sample size was determined, including all assumptions supporting the sample size calculation	Partially addressed. Methods: Sample size described, no formal calculation.
Recruitment	20	Strategies for achieving adequate participant enrollment to reach target sample size	Addressed. Methods.
**Methods: Assignment of interventions**			
Randomization:			
Sequence generation	21 a	Who will generate the random allocation sequence and the method used	Not applicable. Study design: Non-randomised design.
	21 b	Type of randomization (simple or restricted) and details of any factors for stratification. To reduce predictability of a random sequence, other details of any planned restriction (e.g., blocking) should be provided in a separate document that is unavailable to those who enroll participants or assign interventions	Not applicable – Study design.
Allocation concealment mechanism	22	Mechanism used to implement the random allocation sequence (e.g., central computer/telephone; sequentially numbered, opaque, sealed containers), describing any steps to conceal the sequence until interventions are assigned	Not applicable – Study design.
Implementation	23	Whether the personnel who will enroll and those who will assign participants to the interventions will have access to the random allocation sequence	Not applicable – Study design.
Blinding	24 a	Who will be blinded after assignment to interventions (e.g., participants, care providers, outcome assessors, data analysts)	Not applicable – Study design: No blinding.

	24 b	If blinded, how blinding will be achieved and description of the similarity of interventions	Not applicable – Study design.
	24 c	If blinded, circumstances under which unblinding is permissible, and procedure for revealing a participant's allocated intervention during the trial	Not applicable – Study design.
**Methods: Data collection, management, and analysis**			
Data collection methods	25 a	Plans for assessment and collection of trial data, including any related processes to promote data quality (e.g., duplicate measurements, training of assessors) and a description of trial instruments (e.g., questionnaires, laboratory tests) along with their reliability and validity, if known. Reference to where data collection forms can be accessed, if not in the protocol	Addressed – Methods – Data collection.
	25 b	Plans to promote participant retention and complete follow-up, including list of any outcome data to be collected for participants who discontinue or deviate from intervention protocols	Not applicable – Study design: No follow-up beyond hospital stay.
Data management	26	Plans for data entry, coding, security, and storage, including any related processes to promote data quality (e.g., double data entry; range checks for data values). Reference to where details of data management procedures can be accessed, if not in the protocol	Addressed – Methods – Data management.
Statistical methods	27 a	Statistical methods used to compare groups for primary and secondary outcomes, including harms	Addressed – Methods – Statistical analysis.
	27 b	Definition of who will be included in each analysis (e.g., all randomized participants), and in which group	Addressed – Methods – Statistical analysis.
	27 c	How missing data will be handled in the analysis	Addressed – Methods – Statistical analysis.
	27 d	Methods for any additional analyses (e.g., subgroup and sensitivity analyses)	Addressed – Methods – Statistical analysis.
**Methods: Monitoring**			
Data monitoring committee	28 a	Composition of data monitoring committee (DMC); summary of its role and reporting structure; statement of whether it is independent from the sponsor and funder; conflicts of interest and reference to where further details about its charter can be found, if not in the protocol. Alternatively, an explanation of why a DMC is not needed	Not applicable – Study design.
	28 b	Explanation of any interim analyses and stopping guidelines, including who will have access to these interim results and make the final decision to terminate the trial	Not applicable – Study design.
Trial monitoring	29	Frequency and procedures for monitoring trial conduct. If there is no monitoring, give explanation	Not applicable – Study design.
**Ethics**			
Research ethics approval	30	Plans for seeking research ethics committee/institutional review board approval	Addressed. Ethics

Protocol amendments	31	Plans for communicating important protocol modifications to relevant parties	Not applicable. Not described
Consent or assent	32 a	Who will obtain informed consent or assent from potential trial participants or authorized proxies, and how	Addressed. Ethics
	32 b	Additional consent provisions for collection and use of participant data and biological specimens in ancillary studies, if applicable	Not applicable. Study design
Confidentiality	33	How personal information about potential and enrolled participants will be collected, shared, and maintained in order to protect confidentiality before, during, and after the trial	Addressed .Methods / Ethics
Ancillary and post-trial care	34	Provisions, if any, for ancillary and post-trial care, and for compensation to those who suffer harm from trial participation	Not applicable. Design

*We strongly recommend reading this checklist in conjunction with the SPIRIT 2025 Explanation and Elaboration and the SPIRIT 2025 Expanded Checklist for important clarifications on all the items. We also recommend reading relevant SPIRIT extensions. See www.consort-spirit.org

Citation: Chan A-W, Boutron I, Hopewell S, Moher D, Schulz KF, et al. SPIRIT 2025 statement: updated guideline for protocols of randomised trials. BMJ 2025;389:e081477. https://dx.doi.org/10.1136/bmj-2024-081477

© 2025 Chan A-W et al. This is an Open Access article distributed under the terms of the Creative Commons Attribution License (https://creativecommons.org/licenses/by/4.0/), which permits unrestricted use, distribution, and reproduction in any medium, provided the original work is properly cited.
